# In silico model of basal ganglia deep brain stimulation in Parkinson’s disease captures range of effective parameters for pathological beta power suppression

**DOI:** 10.1371/journal.pcbi.1013280

**Published:** 2026-02-11

**Authors:** Mahboubeh Ahmadipour, Federico Fattorini, Nicolò Meneghetti, Alberto Mazzoni

**Affiliations:** 1 The Biorobotics Institute, Scuola Superiore Sant’Anna, Pisa, Italy; 2 Department of Excellence for Robotics and AI, Scuola Superiore Sant’Anna, Pisa, Italy; Universiteit Twente, NETHERLANDS, KINGDOM OF THE

## Abstract

In Parkinson’s disease (PD), the beta band (12–30 Hz) component of basal ganglia activity is pathologically high. Deep brain stimulation (DBS) is an effective treatment to suppress symptoms of PD and is known to suppress pathological beta activity. However, the mechanism underlying this effect is not completely understood. Here, we tested the circuital effects of DBS in a computational model of the basal ganglia network in dopamine-depleted condition mimicking PD. Our model reproduces suppression of beta pathological oscillations in the basal ganglia network induced by subthalamic nucleus (STN) DBS. Crucially, this occurs for realistic levels of DBS intensity only if we incorporate short-term plasticity in projections from STN, increasing the DBS intensity required for suppression of pathological beta oscillations. STN stimulation hampers beta oscillations in the subthalamo-pallidal beta loop. This induces a progressive dephasing between this loop and the striato-pallidal beta loop, which leads in turn to a network-wide suppression of beta oscillations. This is also reflected in a restoration of the balance between D1 and D2 firing rates, which was altered by dopamine depletion. Moreover, the model also reproduces the DBS-induced gamma activity associated with symptom recovery. Finally, we explored the circuital effects of a broad range of DBS parameters in suppressing beta oscillations, focusing on the clinically relevant range of 60–150 Hz stimulation. The model suggests that 60–80 Hz stimulation frequencies might achieve beta desynchronization for an intensity even lower than the one needed at the standard 130 Hz frequency. Overall, our model lays the ground for in-silico tests of a broad spectrum of stimulation patterns.

## Author summary

Deep brain stimulation (DBS) is an effective treatment for alleviating symptoms of Parkinson’s disease (PD), but its underlying mechanism is unclear. Motor symptoms of PD are linked to exaggerated beta-band oscillations in a particular brain area called basal ganglia (BG). In this study, we developed a computational model to simulate how DBS affects brain circuits involved in PD. We found that stimulation can reduce pathological beta rhythms at realistic intensities only when we include realistic synaptic dynamics that temporarily adjust connection strength based on recent activity. Our model shows that stimulation decouples two available beta loops in the BG network and helps restore normal patterns of activity. Interestingly, we also found that stimulation at 60–80 Hz might be effective at lower intensities than the standard 130 Hz. Our work provides new insights into how DBS functions and supports safe and efficient exploration of a wide range of stimulation patterns.

## Introduction

Parkinson’s disease (PD) is the second most common neurodegenerative disorder, predominantly recognized for its motor symptoms, including tremor at rest, rigidity, bradykinesia, akinesia, and postural instability [1]. Beyond motor impairments, PD also encompasses a spectrum of non-motor symptoms, such as sleep disturbances, constipation, mood disorders, autonomic dysfunction, and fatigue [2]. Motor symptoms are associated with loss of dopaminergic neurons in the substantia nigra pars compacta (SNc) and consequent alterations in neural dynamics of basal ganglia (BG) [3–5]. Dopamine depletion destroys the balance in striatal activity by increasing the firing rate of dopamine-inhibited D2 neurons and decreasing the firing rate of dopamine-excited D1 neurons [4], with pathological beta oscillations (12–30 Hz) subsequently emerging in the striatum, subthalamic nucleus (STN), globus pallidus pars externa (GPe), and globus pallidus pars interna (GPi) [6–11]. Several studies investigated the generation mechanism through which dopamine depletion leads to these pathological oscillations in both experimental [12–15] and computational works [16–23]. The first hypothesis suggests that beta oscillations emerge from the interaction between GPe and STN [12,16,17], while other studies propose that the cortex [18–20] or striatum [21] may be the origin. Another hypothesis emphasizes the significant contribution of the interaction between GPe and the striatum in driving these oscillations [13–15,22]. In our previous study, we suggested that the synchronization of two loops (STN-GPe loop and GPe-striatum loop) that are independently oscillating in the beta range is the key factor responsible for these abnormal oscillations [23]. For high levels of dopamine, the two loops are decoupled, and the oscillation power is low while with dopamine depletion the two loops synchronize and produce the pathological oscillations.

Deep brain stimulation (DBS) is a neuromodulation technique which alleviates motor symptoms of PD, but its mechanism is not clear yet. One key hypothesis is local suppression, where somatic firing is reduced via mechanisms like GABAergic activation, synaptic depression, or depolarization blockade [24–26]. Several studies investigated the downstream, and their findings were contrary to what was expected according to suppression of target area since STN-DBS increased pallidal firing [27] despite the excitatory STN-pallidal projections and pallidal DBS reduced thalamic firing [28] despite the inhibitory pallidal-thalamus connections. Therefore, it was proposed that while somatic firing may be suppressed, efferent axons and synaptic terminals are activated at stimulation frequency, leading to the axon-soma decoupling hypothesis [29,30]. An alternative explanation is that DBS activates the afferent axons converging on target neurons. According to this explanation, the distribution of afferent inputs (whether mainly inhibitory or excitatory) and the local neuroanatomical features would determine whether DBS produces a net local inhibitory or excitatory response [31]. Additionally, it was hypothesized that DBS induces antidromic (toward cortex) action potential propagation [25,32]. In addition to modulating average firing rate, DBS significantly alters the oscillatory dynamics in PD: Numerous studies have shown that high-frequency stimulation reduces exaggerated beta-band oscillations, which are closely linked to motor impairments in PD [33–37]. DBS has also been shown to enhance gamma band activity, which is often associated with improved motor performance [33,38]. Gamma oscillations are not specific to DBS, as they have been consistently observed across BG structures in PD patients during dopaminergic treatment [39–41], where they are dynamically modulated by movement [42,43]. This supports a prokinetic role of gamma synchronization, facilitating voluntary movement and alleviating bradykinesia. However, excessive or spatially widespread gamma synchronization has also been linked to levodopa-induced dyskinesias [44–46]. This apparent dual role, promoting movement when physiologically expressed but contributing to involuntary hyperkinesia when abnormally synchronized across the BG, thalamus and cortical network, suggests that the functional impact of gamma activity depends on the degree of coherence within the circuit and its overall dynamical state [44,45].

To better understand the mechanisms underlying DBS effects, computational models have been developed to simulate BG circuits under healthy, Parkinsonian, and DBS conditions [46–55], ranging from detailed spiking neuronal networks [46–53,55] to mean-field approaches [54]. One notable limitation of most current modeling studies is their omission of short-term plasticity (STP), which can play a critical role in shaping neural circuit dynamics [56], in particular at the high firing frequency induced by DBS. Experimental reports of stimulation-induced STP within BG circuits further motivate its consideration [57–60]. Crucially, experimental and modeling work on pallidal circuits demonstrates that STP at STN to GP synapses exhibit a characteristic temporal profile: facilitation for the first few pulses of a high-frequency train followed by a shift to depression as the train continues [61]. This frequency-dependent transition directly alters the postsynaptic response to repeated activation and therefore has clear implications for interpreting and reproducing DBS-compatible network effects. Motivated by these findings, in this study, we extended our previously established BG model [23] by incorporating the biophysical Hanson and Jaeger synaptic plasticity formulation [61] to investigate the impact of STN-DBS on BG dynamics under Parkinsonian conditions. We modeled the effect of STN-DBS as activation of efferent axons close to DBS electrode. Our simulations showed that STN-DBS suppresses beta oscillations in BG and enhances gamma oscillations in striatal medium spiny neurons (MSNs). In addition, we investigated how STN-DBS can suppress beta activity and the effect of stimulation parameters on the beta power. The results revealed that STN-DBS desynchronizes the two principal loops which are responsible for the generation of beta oscillations.

## Results

### Interplay Between Stimulation Intensity and Synaptic Plasticity in DBS-Induced Beta Power Suppression

**Fig 1:**
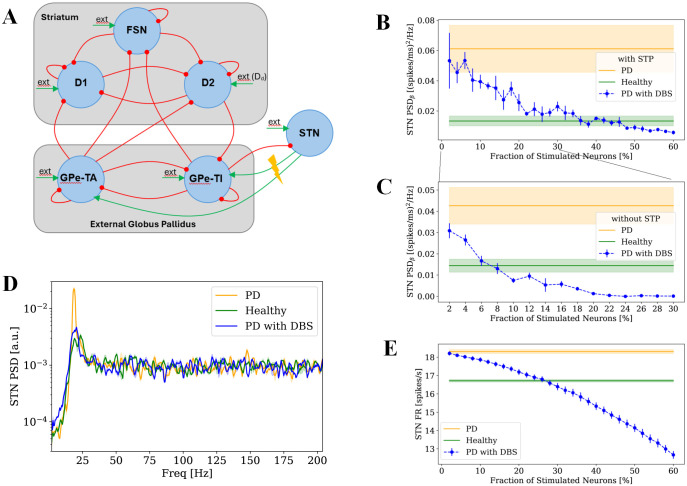
Effect of STN-DBS on STN. **(A)** Architecture of the spiking BG network model with DBS applied to the STN. FSN, D1 and D2: striatal fast spiking neurons, and medium spiny neurons with D1 and D2 dopamine receptors; GPe-TA and GPe-TI: arkypallidal and prototypic populations of the globus pallidus pars externa; STN: subthalamic nucleus; ext: external excitatory Poissonian input. Red/green arrows indicate inhibitory/excitatory projections. Dopamine depletion (D_d_, external drive) was modeled by adjusting the excitatory Poissonian input to D2 neurons. DBS effect was modeled by replacing the output from a fraction of STN neurons to the connected GPe neurons with an imposed TTL signal simulating DBS pattern. **(B)** Variation of STN beta power in relation to fraction of stimulated neurons in BG network model with plasticity. **(C)** Variation of STN beta power in relation to fraction of stimulated neurons in BG network model without plasticity. **(D)** PSD of STN in healthy (green), Parkinsonian (orange) and Parkinsonian with STN-DBS at 40% intensity (blue) conditions in BG network model with plasticity. PSDs are presented as normalized mean±standard error (each divided by its total power) across four simulations. **(E)** Variation of STN firing rate in relation to fraction of stimulated neurons in BG network model with plasticity. For each condition in **(B-E)**, four BG network realizations were simulated; healthy, Parkinsonian and DBS conditions are presented in green, orange, and blue, respectively. For **(B**, **C**, **E)**, results are presented as mean values across four simulations with shaded areas for healthy and Parkinsonian conditions and error bars for the DBS conditions, both representing standard error.

We investigated whether a simple model of STN-DBS effects on a spiking network model of dopamine-depleted BG (see Methods and Fig 1A) is able to replicate the neural activity modulations induced by DBS on STN observed in experiments. Each simulation was run for 6000 ms and the first 2000 ms was eliminated as of the transition state. Baseline DBS effects were first examined using an inter-pulse interval of $t_{DBS}=\text{7}\;ms$ corresponding to stimulation frequency of 143 Hz. It is well known that PD related dopamine depletion leads to aberrant beta power in the STN, and this effect is captured by our model (compare orange and green line in Fig 1B). The most relevant effect of DBS on STN is the decrease in beta range power as a function of the stimulation intensity. Indeed, in our model, progressively increasing the number of neurons activated by DBS (see Methods) results in the restoration of levels of beta power similar to the ones of the non-dopamine depleted network (Fig 1B). Stimulating approximately 40% of STN neurons was necessary to suppress beta activity to normal levels, aligning with clinical observations that indicate activation of an adequate volume of neurons is essential for achieving therapeutic benefits [62,63].

We examined the effect of DBS duration on beta suppression in both models with and without STP (S1 Fig). Our findings showed that the duration of 6000 ms corresponds to a stationary point: increasing or decreasing the stimulation duration did not lead to meaningful changes in beta power. These results indicate that the chosen stimulation duration is sufficient to capture the steady-state effect of DBS on beta oscillations.

Then, we tested the robustness of these results to changes in plasticity parameters by varying the time constants governing short-term facilitation and depression by ±25% for all synapse types. The stimulation fraction required to achieve successful DBS remained consistent across these variations, demonstrating that our findings are not dependent on the specific choice of plasticity time scales (S2 Fig). Next, we investigated the contribution of each type of plastic synapses (i.e., facilitation dominant, depression dominant and pseudo-linear) to DBS efficacy by repeating the analysis in networks with just one type of synapse (S3 Fig). Models containing only facilitation-dominant or pseudo-linear synapses yielded outcomes comparable to the reference model. In contrast, depression-dominant synapses prevented beta oscillation generation in dopamine-depleted conditions, rendering DBS irrelevant in modulating network beta activity. This is explained by the fact that depression-dominant synapses converge to lower steady-state weights, substantially weakening the STN-GPe-TI projection, which is known to be necessary for beta oscillation generation [23].

When synaptic plasticity was not included, the model produced an unrealistic outcome: stimulating only 8% of STN neurons was sufficient to eliminate excessive beta power and restore normal levels (Fig 1C). This contradicts experimental and clinical findings, where DBS typically requires a broader stimulation of the STN neurons to effectively reduce pathological symptoms [64,65]. This discrepancy suggests that synaptic plasticity within the BG network plays a crucial role in shaping the effects of DBS and must be accounted for to develop realistic models of PD. Further increasing DBS stimulation in the BG network model without plasticity resulted in a quick suppression of STN activity (S4 Fig).

To further determine whether synaptic plasticity is required at both GPe subpopulations, we repeated the analysis by selectively disabling plasticity in the STN-to-GPe-TI and STN-to-GPe-TA projection (S5 Fig). We found that when plasticity was included only in the STN-to-GPe-TI projections, beta suppression was comparable to that of the full model, whereas plasticity only at STN-to-GPe-TA resulted in a beta suppression comparable to the model without STP (S5A Fig).

This effect can be explained by examining STN firing activity (S5B Fig). Removing plasticity in the entire model or specifically at STN-to-GPe-TI synapses, caused a strong suppression of STN firing rates, which in turn led to beta suppression at low stimulation intensities. Introducing plasticity limits the efficacy of DBS on the STN-to-GPe-TI projection, reducing pallidal inhibition onto STN and thereby preventing excessive suppression of STN activity. Together, these results indicate that STP at STN-to-GPe-TI synapses is crucial for shaping DBS effects and highlight the central role of the pallidal prototypic population in mediating DBS-induced beta suppression.

Considering the stimulation of 40% of neurons as an effective DBS condition, we compared the power spectral densities (PSDs) across all conditions, finding no other significant alterations in the overall STN spectrum (Fig 1D). Increasing the fraction of stimulated neurons decreased the STN firing rate (Fig 1E) which is in line with a group of experimental results that report the inhibitory effect of DBS in target area [66–68].

Finally, we assessed whether our results were altered by considering the synaptic alterations induced by dopamine depletion ([69], see Methods), along with the increased excitability of D2 populations already implemented in the model. We computed the beta power for 500 simulations with different values of $D_{d}\;$ and $\phi (\alpha_{d})$ (the parameter regulating the synaptic alterations, see Methods). We identified several combinations of these parameters that produced beta power comparable to that of our original model (S6A Fig). As expected, both modifications increased beta oscillations under dopamine depleted condition. We selected an intermediate combination, $D_{d}=\text{0}.\text{3}\;$ and $\varphi (\alpha_{d})=-\text{0}.\text{4}$, and we repeated the analysis shown in Fig 1B, evaluating the effect of DBS intensity on beta power (S6B Fig). The model incorporating synaptic alterations displayed trends consistent with the original model; however, a larger fraction of stimulated neurons was required to achieve comparable levels of beta suppression, suggesting that synaptic alterations render pathological synchronization more robust to DBS. Nevertheless, both models demonstrated the effectiveness of DBS in suppressing beta oscillations, showing that this effect can be reproduced independently of the specific mechanism through which dopamine depletion leads to pathological synchronization.

### DBS modulates neural activity spectrum across BG: Beyond beta suppression

**Fig 2:**
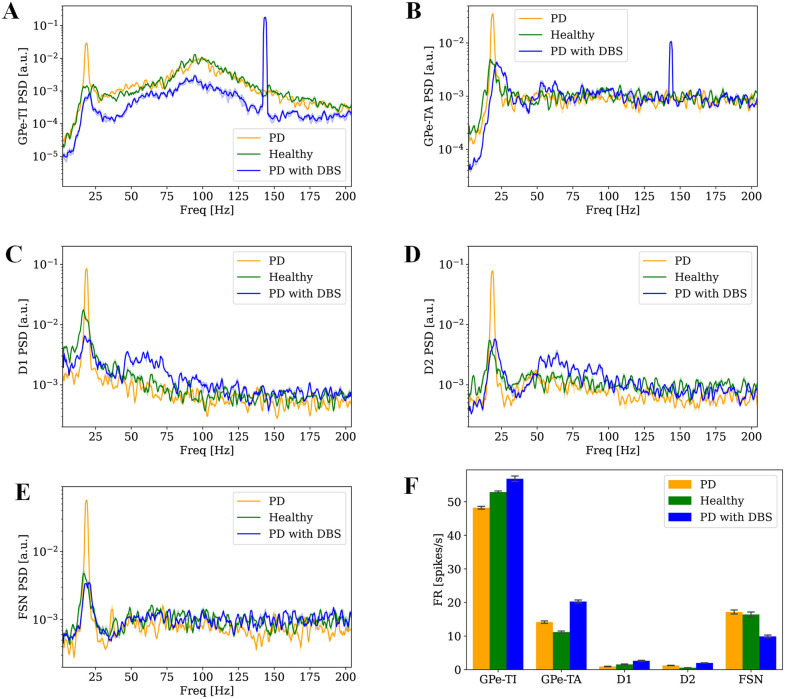
STN-DBS induced spectral changes across nuclei. PSDs of GPe-TI **(A)**, GPe-TA **(B)**, D1 **(C)**, D2 **(D)** and FSN **(E)** in healthy (green), Parkinsonian (orange) and Parkinsonian with STN-DBS at 40% intensity (blue) conditions. PSDs are presented as normalized mean±standard error (each divided by its total power) across four simulations. **(F)** Firing rate of BG nuclei in healthy (green), Parkinsonian (orange) and Parkinsonian with STN-DBS at 40% intensity (blue) conditions. Firing rates are presented as mean values across four simulations, with error bars indicating standard deviations.

Building on the findings from the STN-DBS effect on STN, we next explored its network-level impact on the other BG nuclei. While these regions, like STN, showed reduced beta power, the effects of STN-DBS on them extend also over other frequency ranges (Fig 2). GPe-TI and GPe-TA directly receive DBS pulses from STN axons and displayed sharp peaks near the stimulation frequency along with reductions in beta power of 73.1% and 70.8%, respectively (Fig 2A-B), whereas such a peak is absent in the other nuclei that do not receive direct STN input (Fig 2C-E). STN-DBS decreased beta activity in D1 and D2 while it enhanced their gamma activity (Fig 2C-D). In our previous work [70], we assessed the statistical significance of gamma oscillations using a threshold-based criterion: the spectral power in the gamma range was compared to that of Poisson processes with the same mean firing rate, and an oscillation was considered significant when the mean power exceeded this threshold. Consistent with this, gamma-band activity was absent in D1 neurons under healthy and Parkinsonian conditions, but emerged following STN-DBS (Fig 2C), showing a 555% increase. In addition, STN-DBS led to a 58.9% decrease in beta activity in D1 neurons. In contrast, D2 neurons exhibited gamma oscillations under PD condition even prior to stimulation (Fig 2D). With STN-DBS, beta activity in D2 neurons decreased by 78.9%, accompanied by a 133.7% increase in gamma band activity (Fig 2D). Finally, the behavior of FSN was similar to the one observed in STN (Fig 2E). STN-DBS altered the firing rates of these structures in Parkinsonian state (Fig 2F). GPe-TI and GPe-TA neurons exhibited distinct responses to dopamine depletion. GPe-TI neurons showed a reduction in mean firing rate, consistent with their strong inhibitory input from D2 MSNs, whose activity is enhanced in the dopamine-depleted state. Conversely, GPe-TA neurons displayed an increased firing rate, reflecting their differential connectivity and reduced inhibitory drive. Under STN-DBS, instead, the firing rates of both pallidal subpopulations increased markedly (by 17.9% for GPe-TI and 43.2% for GPe-TA), consistent with the stronger excitatory input from STN afferents entrained by stimulation. STN-DBS resulted in varied modulation of neuronal subtypes within the striatum. Firing rates of D1 and D2 neurons increased by 183.3% and 59.2%, respectively, while FSN exhibited 42.3% reduction in activity. STN-DBS restored the balance in striatal MSNs by increasing the firing rate of D1 neurons relative to D2 neurons, thereby resembling the healthy state.

Beyond these frequency-specific effects, our simulations indicate that STN-DBS does not generate novel resonant dynamics in the BG. Instead, stimulation modulates oscillatory components that are already present in the healthy and Parkinsonian circuits. In the beta range, DBS consistently reduced power across all nuclei. In the gamma range, DBS revealed distinct nucleus-specific patterns: gamma emerged in D1 neurons only under stimulation, whereas in D2 and GPe-TI neurons DBS enhanced pre-existing gamma components. This pattern mirrors the intrinsic oscillatory tendencies embedded in the network architecture [70], which become more prominent as STN-DBS increases the firing rates of these inhibitory populations. Thus, the oscillatory signatures observed under DBS reflect the modulation of existing resonant properties rather than the emergence of stimulation-induced rhythms.

In addition, raster plots and instantaneous population firing rates of all BG structures were presented in S7 Fig, providing a detailed visualization of their temporal modulation under 40% intensity of STN-DBS. DBS onset induces a transient transition state in the BG network around 200 ms. STN and FSN activities were initially strongly suppressed and subsequently increase. GPe-TI reaches its maximum activity and then decreases, and GPe-TA, D1, and D2 activities rise and subsequently decrease.

### DBS desynchronizes the two beta oscillators in BG

**Fig 3:**
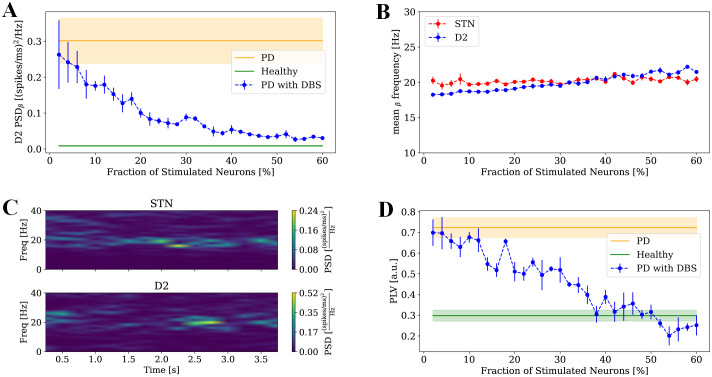
DBS-induced desynchronization of beta oscillators in BG. **(A)** D2 beta power in healthy (green), Parkinsonian (orange), and Parkinsonian with STN-DBS (blue), with the blue curve also showing its variation with the fraction of stimulated STN neurons. **(B)** Mean beta frequencies of STN (blue) and D2 (red) in relation to the fraction of stimulated neurons in STN. Results are reported as mean and standard error across four simulations. **(C)** Spectrogram of STN (top) and D2 (bottom) in Parkinsonian with STN-DBS at 40% intensity. **(D)** PLV between STN and D2 in healthy (green), Parkinsonian (orange), and Parkinsonian with STN-DBS (blue), with the blue curve also showing PLV variation with the fraction of stimulated STN neurons. For **(A, D)**, results are presented as mean values across four simulations with shaded areas for healthy and Parkinsonian conditions and error bars for the DBS conditions, both representing standard error.

To better understand how DBS suppresses pathological beta oscillations, we focused on the two principal beta-generating loops in the BG. Our previous study identified the STN–GPe-TI loop and the FSN–D2–GPe-TI loop as independent beta oscillators under healthy dopamine levels, which progressively synchronize as dopamine is depleted, contributing to the emergence of pathological beta oscillations in PD [23]. Our findings in the previous sections indicated that beta power in the STN decreased as the stimulation intensity increased (Fig 1B). To understand the DBS effect on the GPe-striatum loop, we studied the effect of STN-DBS on the beta power of D2. Similar to our findings for STN, beta power in D2 decreases as stimulation intensity increases and when approximately 40% of STN neurons are stimulated, D2 beta power approaches the beta power level in the healthy condition (Fig 3A). Mean beta frequencies (i.e., spectral centroids of the PSDs within the 12–30 Hz band; see Methods) of STN and D2 were close for all stimulation intensities tested (Fig 3B). The spectrograms of the STN and D2 at 40% stimulation of STN neurons showed that the two oscillators were not synchronized even with very similar mean beta frequencies (Fig 3C). To quantify the synchronization of the two oscillators in different conditions, the phase locked variable (PLV) was then computed, and the results demonstrated that at high levels of dopamine (healthy condition) the two oscillators were highly independent (PLV≅0.3) while at low levels of dopamine (PD condition), they were highly synchronized (PLV≅0.7) (Fig 3D). As DBS intensity increased, the synchronization of the two oscillators decreased and at around 40% stimulation of STN neurons, the PLV reached the PLV value in healthy condition (Fig 3D). Considering the model with the synaptic alterations induced by dopamine depletion resulted in similar trend for the PLV (Methods and S6C Fig). However, as for beta power, a larger fraction of stimulated neurons (around 50%, rather than 40%) was required to achieve healthy levels of PLV.

### Impact of DBS settings on beta power modulation

**Fig 4:**
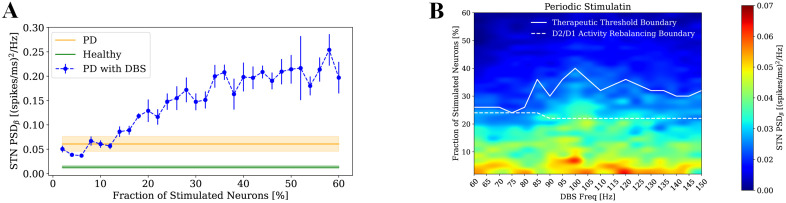
Effects of DBS settings on STN beta power. **(A)** STN beta power in healthy (green), Parkinsonian (orange), and Parkinsonian with Poissonian STN-DBS (blue), with the blue curve also showing its variation with the fraction of stimulated STN neurons. Results are presented as mean values across four simulations with shaded areas for healthy and Parkinsonian conditions and error bars for the DBS conditions, both representing standard error. **(B)** STN beta power in relation to DBS frequency and intensity. The white boundary line indicates the therapeutic threshold, representing the minimum stimulation intensity needed at each frequency to bring beta power within the healthy range. The dashed white line indicates the boundary for D2/D1 activity rebalancing, representing the minimum stimulation intensity needed at each frequency to reduce the D2/D1 firing-rate ratio below 1. Results are presented as mean values across four simulations.

The results in the previous sections showed that the model can capture the main acute effects of the conventional DBS with 7 ms inter-pulse interval on BG activity. For further exploration, we studied the impact of DBS settings on DBS outcome. First, we studied the influence of DBS temporal structure on the modulation of beta power by considering Poissonian inputs as DBS pulses. Our simulations showed that delivering DBS as a Poisson process with an average inter-pulse interval of 7 ms was not able to suppress beta power in STN (Fig 4A). Indeed, the STN beta power increased as the Poissonian DBS intensity increased. This result suggests that to effectively disrupt the strong pathological beta rhythm and to restore normal BG network dynamics, a regular input is necessary. In contrast, the Poissonian input acts as white noise and is not effective in destroying the beta rhythm. Then, we investigated the impact of stimulation parameters including intensity and frequency on pathological oscillatory activity of STN (Fig 4B). Stimulation frequency was varied from 60 Hz to 150 Hz in 5 Hz increments, while intensity was defined as the fraction of STN neurons receiving stimulation, ranging from 2% to 60% in 2% steps. A heatmap was generated to visualize STN beta power across this parameter space and a white boundary line was overlaid on the plot to indicate the therapeutic threshold- the minimum stimulation intensity required at each frequency to reduce beta power to within the healthy range. Stimulation intensities above this boundary maintained beta power at or below the healthy level, indicating effective and stable therapeutic control. Interestingly, the therapeutic boundary reveals a non-monotonic pattern: stimulation frequencies in the range of approximately 85–125 Hz require relatively higher stimulation intensities to reduce beta power to the healthy level. In contrast, both lower frequencies (below ~80 Hz) and higher frequencies (above ~130 Hz) are associated with reduced intensity requirements. This suggests that frequencies outside the mid-range (85–125 Hz) may be more efficient in suppressing pathological beta activity, potentially reflecting complex interactions between stimulation dynamics and BG network across the frequency spectrum. This analysis provides insight into the combined role of frequency and intensity in achieving optimal DBS outcomes, supporting the use of high-frequency stimulation for Parkinsonian symptom relief, while also aligning with clinical observations [71] suggesting potential benefits of lower frequency stimulation (60–80 Hz).

In addition, Fig 4B includes the boundary for D2/D1 activity rebalancing, defined as the minimum stimulation intensity at each frequency required to reduce the D2/D1 firing-rate ratio below 1. For stimulation intensities above this boundary, the ratio is below 1 reflecting a substantial shift toward the healthy balance (mean: 0.37) relative to the pathological state (mean: 1.36). This boundary lies consistently below the therapeutic threshold for beta suppression, indicating that rebalancing D2/D1 activity occurs at lower stimulation intensities than those required for full beta power normalization. These findings suggest that DBS parameters above the therapeutic boundary not only suppress pathological oscillations but also robustly promote a D2–D1 activity balance closer to the healthy regime, highlighting the stability and robustness of the therapeutic effect.

## Discussion

In this study, we presented a computational model of the effects of STN-DBS on the BG network. The model reproduces DBS-induced beta suppression and highlights the role of dephasing between the two beta loops in the process. Moreover, we found that STN-DBS enhances gamma band activity in the striatum, particularly in D1 and D2 receptor-type MSNs. These results are consistent with previous experimental studies showing that DBS reduces beta oscillations [33–37] and enhances gamma activity [33,38] in the BG.

In clinical practice, DBS intensity is adjusted through the stimulation amplitude and pulse width, which jointly determine the volume of tissue activated (VTA) around the electrode. In our model, the fraction of stimulated neurons provides a conceptual representation of this VTA, with higher fractions corresponding to larger activation volumes induced by higher amplitudes or longer pulse widths. Clinically, the amplitude is gradually increased during postoperative programming to identify the threshold at which therapeutic benefit appears and the threshold at which stimulation-induced side effects emerge, with the final setting chosen within this therapeutic window. DBS amplitude may be refined further over time along with pulse width and frequency adjustments to maintain optimal symptom control. In our model, beta power was used as a biomarker of Parkinsonian state. Therefore, the fraction of stimulated neurons that reduced beta power to healthy levels was interpreted as an effective stimulation intensity. We found that beta suppression is achieved for realistic ranges of intensity only if STP is taken into account. The observed beta suppression in our model is dependent on stimulation intensity, with a threshold of approximately 40% of STN neurons required to be stimulated to achieve normalization of beta power. This finding is consistent with clinical observations that sufficient stimulation volume is necessary to attain therapeutic effects [62,63]. At this level of recruitment, two main network changes occur: (1) the mean firing rate of STN neurons, which is abnormally elevated in the Parkinsonian state, decreases (Fig 1E), and (2) the excessive phase locking between the striato-pallidal (FSN–D2–GPe-TI) and pallido-subthalamic (STN–GPe-TI) beta-generating loops markedly diminishes, restoring PLV comparable to the healthy state (Fig 3D). This desynchronization between the two beta-rhythmogenic loops represents the underlying mechanism responsible for the reduction of pathological beta activity in the model (Fig 1B). Instead, if we do not include STP in our model, beta suppression is achieved with an unrealistic minimal stimulation. According to experimental data, high-frequency stimulation suppresses STN [24–26] and causes high-frequency projections to go downstream through axon terminals [27,28]. Given that DBS affects axonal terminals through repetitive high-frequency activation, accurately capturing synaptic dynamics is essential. STP which reflects transient changes in synaptic strength based on recent activity [56,72], is particularly relevant under such conditions. To enhance the physiological realism of our model, we incorporated STP at these projections to capture the dynamic modulation of synaptic efficacy during repetitive stimulation. We used Hanson and Jaeger model [61] to capture STP in the STN to GPe pathway as it is specifically tuned for this connection based on experimental data from this region. However, we expect the main findings to remain consistent when using alternative STP models, provided their parameters are appropriately tuned according to relevant experimental data. To simulate DBS, we modeled high frequency stimulation by replacing the physiological projections from the STN to the GPe with periodic high-frequency pulses, mimicking clinical stimulation patterns.

In the present work, short-term synaptic plasticity was sufficient to reproduce the desynchronizing effects of DBS on pathological beta oscillations. Nonetheless, this does not exclude the potential contribution of long-term synaptic mechanisms, which could act in parallel and shape the network response over extended timescales. Future modeling developments could integrate such long-term forms of plasticity, such as spike-timing–dependent plasticity, to explore how DBS may induce lasting modifications in BG–thalamocortical connectivity, as suggested by recent studies [73–76].

Our model shows that DBS suppresses pathological beta activity by decoupling two interacting beta-generating loops within the BG: the striato-pallidal and pallido-subthalamic circuits (Fig 3D). Increasing stimulation amplitude recruits more STN efferents to GPe, enhancing GPe self-inhibition and weakening inter-loop coupling. While moderate decoupling restores physiological activity, excessive stimulation over-suppresses beta synchrony, driving power below the healthy range (Fig 1B). This “overcorrection” implies that DBS stronger than therapeutical levels may disrupt normal network coordination, consistent with clinical evidence of negative side effects and clinical reduced efficacy at high amplitudes [77–79].

Previous studies primarily focused on how DBS influences thalamic relay fidelity and beta oscillations within the BG network [46–55]. The thalamus is of particular interest because motor impairments in PD are believed to arise from its disability to relay sensorimotor input to the cortex. In the Parkinsonian state, abnormal projection from the output nucleus of BG neuronal network (i.e., GPi) to thalamus are thought to disrupt normal thalamic relay function, leading to impaired motor control. In contrast to much of the modeling work that has centred on thalamic dynamics, Adam et al. [55] investigated the effects of STN-DBS on BG, showing that it can restore gamma band oscillations in the striatum, which are disrupted in the Parkinsonian state. Although these models have provided valuable insights into the effects of DBS, they did not account for STP, leaving a gap in understanding how dynamic synaptic mechanisms contribute to the network’s response to DBS. Taking this into account and modeling DBS by manipulation of projections from STN to GPe showed increased activation of the GPe, particularly the GPe-TI population. Increased activity of GPe in turn exerted stronger inhibition onto the STN, recapitulating the suppressive feedback observed experimentally. However, the effects of DBS in our model extended beyond this local circuit. Neuronal populations that receive direct or indirect input from the STN, including GPe-TA and striatal neurons (D1, D2, FSNs), also exhibited significant changes in firing rates and spectral properties. Notably, STN-DBS restored the balance of striatal MSNs activity by increasing the activity of D1 neurons relative to D2 neurons, thereby approximating the firing pattern observed under healthy conditions.

In our previous study [23], we examined the circuit-level mechanisms underlying beta oscillations in the BG using a computational model. Specifically, we found that the loop between the STN and GPe-TI, as well as the loop involving striatal fast-spiking interneurons, D2 MSNs, and the GPe-TI, exhibit resonances in the beta range. While these loops function largely independently under healthy dopamine levels, their synchronization under dopamine depletion leads to an amplification of beta-band oscillations [23]. In this study, we investigated the impact of DBS on these oscillations. Our results revealed that STN-DBS impacts the synchronization of the two beta loops. STN-DBS desynchronizes these loops, with the PLV dropping to levels comparable to the healthy state when stimulation intensity reaches 40%. This supports the hypothesis that pathological synchronization of independent beta oscillators contributes to the emergence of abnormal beta rhythms in PD [23] and that DBS restores normal BG function by disrupting this synchronization. Our findings highlight the broad influence of DBS across the BG and underscore the importance of studying BG network as integrated system rather than isolated components.

One of the main objectives of the work was to provide a model enabling in silico testing of DBS parameters. Here we performed a frequency–intensity analysis revealed that achieving therapeutic effects varies non-monotonically across the 60–150 Hz range, with lower stimulation intensities required at both low (<80 Hz) and high (>130 Hz) gamma frequencies. In contrast, the mid-frequency range (~85–125 Hz), which includes the gamma peak of GPe-TI (~100 Hz), required higher stimulation intensities, suggesting possible interference effects that may reduce therapeutic efficiency within this band. Interestingly, although the standard clinical practice is to deliver DBS at 130–150 Hz, recent results have shown that stimulation at 60 Hz can also be clinically effective [79,80]. Consistently, recent computational work has demonstrated that low-frequency DBS can induce persistent modifications of synaptic connectivity within the GPe–STN loop, leading to a sustained suppression of beta oscillations beyond the stimulation period [81].

Our analysis of stimulation parameters further revealed that Poissonian DBS, which lacks temporal regularity, failed to reduce beta activity and in fact exacerbated it while a regular stimulation like the periodic pulses of standard DBS effectively reduces beta activity. This observation reinforces clinical preferences for high-frequency, regular DBS patterns and suggests that temporal structure plays a key role in disrupting pathological synchrony. It is worth noting that this finding does not conflict with the concept of adaptive DBS (aDBS), in which the stimulation amplitude (rather than its temporal structure) is dynamically adjusted based on the ongoing level of beta activity in STN LFPs [82]. In our model, increasing stimulation amplitude (modeled as the recruitment of a larger fraction of efferent STN axons) produced stronger beta suppression, hence supporting the rationale for adaptive modulation of intensity in aDBS. Furthermore, the timescale of parameter adjustments in clinical aDBS, typically ranging from hundreds of milliseconds to several minutes [83], is compatible with the STP dynamics implemented in our model. Taken together, these results suggest that while effective DBS requires temporally regular stimulation to disrupt pathological synchrony, dynamic adaptation of stimulation amplitude, as in aDBS, can further optimize therapeutic efficacy within this framework.

Despite the strong alignment of our results with experimental studies, our computational framework has certain limitations. Although the model incorporates key BG structures and STP, it simplifies other aspects such as heterogeneous neuronal subtypes, and external inputs. In particular, inputs to all BG populations were modeled as uncorrelated Poisson processes, which do not fully capture their characteristics. Experimental evidence indicates that beta and gamma oscillations in the BG may, in part, be inherited from cortical activity [84–86]. Future studies could enhance biological plausibility by integrating long-term plasticity, detailed cortical and thalamic inputs, and more complex neuronal dynamics to better capture the contributions of these structures to BG function. Another limitation is that we represented dopamine loss by increasing the firing rates of D2 neurons population, capturing the enhanced activity of the indirect pathway characteristic of PD. While this method successfully reproduces key pathological features, it simplifies the broader impact of dopamine depletion by excluding other important factors such as alterations in synaptic strength, plasticity mechanisms, and network-level adaptations [69]. By focusing on the primary consequence of dopamine loss, the imbalance between the direct and indirect pathways of the BG, we were able to replicate major aspects of BG dynamics in PD. Furthermore, it is important to note that experimental evidence on beta oscillations mainly comes from LFP recordings, whereas our simulations produce population activity derived from firing rates. The biophysical basis of LFP generation is well established in the cortex: the geometric alignment of pyramidal neurons produces a coherent spatial organization of transmembrane currents, which gives rise to macroscopic extracellular potentials that can be quantitatively reconstructed as weighted sums of these currents [87–91]. Extending this biophysical framework to the BG is however not straightforward. The STN lacks recurrent connectivity and exhibits a predominantly symmetric neuronal morphology, two features that have been shown to impair the reconstruction of extracellular potentials from modeled transmembrane currents [90,92]. As a result, defining and interpreting LFPs in the STN remains an open theoretical problem. Future modeling work should incorporate this constraint to link simulated neuronal dynamics with experimentally measured field potentials in a biophysically grounded manner.

Of note, we did not include the BG output nuclei (GPi or substantia nigra pars reticulata – SNr) in the present simulations because they act mainly as feed-forward outputs to thalamus and do not provide direct feedback to the BG populations implicated in beta generation; therefore, their omission does not affect our conclusions about local rhythm generation and DBS-mediated modulation. Nonetheless, adding GPi and SNr in future work would allow quantifying how DBS-driven changes are translated to thalamic/cortical output and testing how modulation of BG outputs affects information transfer to motor targets and behavior.

Another important consideration is that our model represents DBS intensity by varying the fraction of STN axons recruited, an approach supported by previous computational studies [93–95]. However, we did not consider the spatial dimension of STN neuron recruitment, which represents a limitation of the current approach. Adding spatial structure in future work could provide further insights into how selective STN subpopulation activation affects downstream activity and DBS outcomes. Finally, since DBS was implemented by replacing the output of a fraction of STN neurons with an imposed stimulation pattern rather than a current-based stimulation waveform [46,52,53,96], our model does not capture pulse waveform or charge-balance-dependent membrane effects, which should be addressed in future work.

In summary, our findings highlight the importance of stimulation intensity and synaptic plasticity in determining the efficacy of STN-DBS. By disrupting pathological synchronization and restoring physiological oscillatory activity, DBS exerts widespread modulatory effects across the BG. These results emphasize the necessity of incorporating biologically realistic synaptic mechanisms in computational models to enhance our understanding of neuromodulation therapies and to guide both the optimization of DBS protocols for PD and the investigation of novel paradigms of stimulation with more complex patterns.

## Materials and methods

### BG network model

We used a spiking neuronal network model of the BG, originally developed in [23] and subsequently updated in [70], which we further modified in this study to incorporate synaptic plasticity mechanisms. The BG network is a comprehensive biophysical model including striatum, GPe, STN and their connections (Fig 1A). The output nuclei of the BG (SNr/GPi) were not included, as they do not provide direct feedback to other BG populations. Therefore, their exclusion does not affect the interpretation of our analysis regarding intrinsic rhythm generation within the BG or how DBS influences its oscillatory dynamics. Striatum consists of D1-type dopamine receptor MSNs (D1-MSN), D2-type dopamine receptor MSNs (D2-MSN) and a population of fast spiking (inter-)neurons (FSN). GPe is divided into two populations that are labelled as GPe-TA (characterized by a lower discharge rate and by a negligible input from striatal populations) and GPe-TI (with a higher activity and receiving input from D2). Each population has a specific size reported in Table 1. Neurons are connected randomly, with fixed connection probabilities between populations, based on previous studies [23,69]. Each neuron receives independent excitatory Poissonian input, simulating afferent signals from other brain regions not explicitly modeled. External input rates were adjusted across populations to align their mean firing rates with ranges reported in murine experimental observations: FSN (10–20 Hz) ([97,98]), D1 and D2 (MSN) (0.5-2.5 Hz) ([99]), GPe-TI (30–60 Hz) ([14,15]), GPe-TA (10–20 Hz) ([15]) and STN (12–20 Hz) ([100]). Population-specific external Poisson input rates were used to achieve realistic baseline firing levels, reflecting differences in effective excitatory synaptic drive rather than assuming distinct anatomical sources.

**Table 1:** Size of populations in BG network.

Population	Number of Neurons
D1	6000
D2	6000
FSN	420
GPe-TA	264
GPe-TI	780
STN	408

Neurons are modeled as conductance based, adaptive and point neurons. Adaptive exponential neurons are applied for the STN and GPe populations and their dynamics are as:


$$\begin{array}{c} C_{m}\frac{dv}{dt}=\;-g_{L}\left(v-E_{L}\right)-g_{ex}\left(v-E_{ex}\right)-g_{in}\left(v-E_{in}\right)+g_{L}\Delta Texp\left(\frac{v-V_{th}}{\Delta T}\right)-w+I_{e}\\ \tau_{w}\frac{dw}{dt}=-w+a\left(v-E_{L}\right) \end{array}$$ (1)

Adaptive quadratic neurons are used for striatal populations; For D1 and D2 populations, the equations are as:


$$\begin{array}{c} C_{m}\frac{dv}{dt}=\;-g_{ex}\left(v-E_{ex}\right)-g_{in}\left(v-E_{in}\right)+k\left(v-E_{L}\right)(v-V_{th})-w+I_{e}\\ \tau_{w}\frac{dw}{dt}=-w+a\left(v-E_{L}\right)\; \end{array}$$ (2)

while for FSN, the dynamics are governed by:


$$\begin{array}{c} C_{m}\frac{dv}{dt}=\;-g_{ex}\left(v-E_{ex}\right)-g_{in}\left(v-E_{in}\right)+k\left(v-E_{L}\right)(v-V_{th})-w+I_{e}\\ \tau_{w}\frac{dw}{dt}=\left\{ \begin{array}{c} \;-w+{a\left(v-V_{b}\right)}^{\text{3}}\;if\;v<V_{b}\\ \;-w\;otherwise\; \end{array}\right.\; \end{array}$$ (3)

In equations (1) to (3), v is the membrane potential, w is the contribution of the neuron’s slow currents and $g_{ex}$ and $g_{in}$ are conductance of excitatory and inhibitory synapses, respectively. For all types of neurons, if the membrane potential reaches $V_{peak}$, it will reset to $V_{reset}$:


$$if\;v>V_{peak}\;then\;v=\;V_{reset},\;w=w+b$$ (4)

In the previous work, all the synapses were considered static while in this work we used frequency-dependent synapses to capture STP for STN to GPe-TI and STN to GPe-TA projections. The change in conductance of static synapses presents an exponential decay:


$$\begin{array}{c} \tau_{ex}\frac{dg_{ex}}{dt}=-g_{ex}+\tau_{ex}\sum_{i}g_{exc.,i}\delta (t-t_{i}) \end{array}$$ (5)

$$\tau_{in}\frac{dg_{in}}{dt}=-g_{in}+\tau_{in}\sum {\mathrm{\backslash nolimits}}_{i}g_{inh.,i}\delta (t-t_{i}-t_{r,i})$$ (6)

For dynamic synapses, we used the model proposed by Hanson and Jaeger that can fit physiological STP data [61]. The idea of the model is to describe facilitation and depression phenomena in synapses. The facilitation mechanism enhances the synaptic strength, and it could reflect the increase in neurotransmitters release due to the calcium influx caused by spikes arriving in the presynaptic terminal [101,102]. The depression mechanism diminishes the synaptic strength due to the consumption of neurotransmitters for the transmission of action potentials. The effective synaptic strength is determined by the combination of both facilitation and depression mechanisms.

The Hanson and Jaeger model uses two variables to describe these phenomena; variables F and D which represent facilitation and depression, respectively. The dynamics of the synapses with STP is governed by:


$$\begin{array}{c} \begin{array}{c} \tau_{F}\frac{dF}{dt}=\text{1}-F+\tau_{F}F({Inc}_{F}-\text{1})\left(\frac{F_{bound}-F}{F_{bound}-\text{1}}\right)\sum_{i}\delta (t-t_{i})\mathrm{\backslash vspace}1mm\\ \tau_{D}\frac{dD}{dt}=\text{1}-D+\tau_{D}D({Inc}_{D}-\text{1})\sum_{i}\delta (t-t_{i})\mathrm{\backslash vspace}1mm\\ \tau_{ex}\frac{dg_{ex}}{dt}=-g_{ex}+\tau_{ex}\sum_{i}g_{ex,i}DF\delta (t-t_{i}) \end{array} \end{array}$$ (7)

where $\tau_{F}$ and $\tau_{D}$ are the time constants of facilitation and depression, respectively. ${Inc}_{F}$ and ${Inc}_{D}$ are the strengths of facilitation and depression, respectively. $F_{bound}$ is an upper bound for the facilitation variable. According to physiological recordings [61], each STN to GPe synapse was designated as one of the three types of plasticity, facilitation-dominant, depression-dominant or pseudo-linear with the same probability.

Parameter values for each neuronal population are reported in Table 2. The connectivity properties (delays (t_r,i_), connection probabilities and synaptic weights (g_exc,i_,g_inh,i_)) are presented in Table 3. These values were acquired from our previous studies [23,70] which has been originated from the work of Lindahl and Kotaleski [69]. All nuclei, except the STN, include self-inhibitory connections. The parameter values of dynamic synapses, which were extracted from Hanson and Jaeger study [61], are listed in Table 4.

**Table 2:** Parameters of each neuronal population.(*): for the FSN population the unit of a is ns/mV^2^. (†): recurrent inhibitory synapses for GPe-TI were modeled separately using a distinct conductance variable with its own time constant $\tau_{rec}=\text{7}$.

Parameter	Unit	Description	D1	D2	FSN	GPe-TI	GPe-TA	STN
$C_{m}$	pF	Membrane capacitance	15.2	15.2	80.0	40.0	60.0	60.0
$E_{L}$	mV	Leak reversal potential	-78.2	-80.0	-80.0	-55.1	-55.1	-80.2
$E_{ex}$	mV	(Excitatory) postsynaptic reversal potential	0.0	0.0	0.0	0.0	0.0	0.0
$E_{in}$	mV	(Inhibitory) postsynaptic reversal potential	-74	-74	-74	-65	-65	-84
$\tau_{ex}$	ms	(Excitatory) synapse time constant	12.0	12.0	12.0	10.0	10.0	4.0
$\tau_{in}$	ms	(Inhibitory) synapse time constant	10.0	10.0	10.0	5.5^†^	5.5	8.0
$V_{th}$	mV	Threshold potential	-29.7	-29.7	-50	-54.7	-54.7	-64
$I_{e}$	pA	Current to obtain *in vitro* firing rate without synaptic input	0.0	0.0	0.0	12.0	1.0	5.0
$V_{reset}$	mV	Spike reset potential	-60	-60	-60	-60	-60	-70
a	nS	Subthreshold adaptation	-20	-20	0.025^*^	2.5	2.5	0.0
b	pA	Spike-triggered adaptation	67.0	91.0	0.0	70.0	105.0	0.05
$\tau_{w}$	ms	Adaptation time constant	100.0	100.0	5.0	20.0	20.0	333.0
$V_{peak}$	mV	Spike cut off potential	40.0	40.0	25.0	15.0	15.0	15.0
ΔT	mV	Slope factor of spike upstroke				1.7	2.55	16.2
$g_{L}$	nS	Leak conductance				1.0	1.0	10.0
k	nS/mV	Steady-state voltage dependence	1.0	1.0	1.0			
$V_{b}$	mV	Voltage dependence recovery current			-55			
$v_{ext}$	kHz	Mean rate of external input	1.12	1.08	0.94	0.82	0.1	0.5

**Table 3:** Connectivity properties of the BG model.

Source	Target	Probability	Delay [ms]	Type	Synaptic Weight [nS]
D1	D1	0.0607	1.7	Inh	0.12
	D2	0.0140	1.7	Inh	0.30
D2	D1	0.0653	1.7	Inh	0.36
	D2	0.0840	1.7	Inh	0.20
	GPe-TI	0.0833	7.0	Inh	1.28
FSN	D1	0.0381	1.7	Inh	6.60
	FSN	0.0238	1.0	Inh	0.50
	D2	0.0262	1.7	Inh	4.80
GPe-TI	GPe-TI	0.0321	1.8	Inh	1.10
	GPe-TA	0.0321	1.8	Inh	0.35
	FSN	0.0128	7.0	Inh	1.60
	STN	0.0385	1.8	Inh	0.08
GPe-TA	D1	0.0379	7.0	Inh	0.35
	D2	0.0379	7.0	Inh	0.61
	FSN	0.0379	7.0	Inh	1.85
	GPe-TA	0.0189	1.8	Inh	0.35
	GPe-TI	0.0189	1.8	Inh	1.20
STN	GPe-TA	0.0735	2.0	Exc	0.13
	GPe-TI	0.0735	2.0	Exc	0.42
ext	D1	1.0	0.0	Exc	0.45
	D2	1.0	0.0	Exc	0.45
	FSN	1.0	0.0	Exc	0.50
	GPe-TI	1.0	0.0	Exc	0.25
	GPe-TA	1.0	0.0	Exc	0.15
	STN	1.0	0.0	Exc	0.25

**Table 4:** Parameter values for STP Synapses.

Synapse Type	$\tau_{F}$(ms)	$\tau_{D}$(ms)	${𝐈𝐧𝐜}_{F}$	${𝐈𝐧𝐜}_{D}$	${𝐅}_{bound}$
Facilitation-dominant	241	491	1.4	0.9	5
Depression-dominant	148	764	1.64	0.55	5
Pseudo-Linear	345	700	1.34	0.86	5

**Fig 5:**
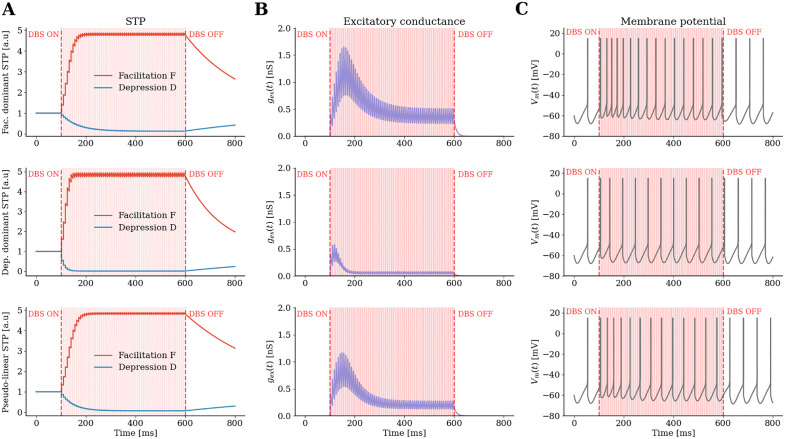
Effect of DBS on three synapse types to GPe-TI neuron: (A) STP variables 𝐃 (depression) and 𝐅 (facilitation) for facilitation-dominant, depression-dominant, and pseudo-linear synapses.Stimulation was delivered with 7 ms inter-pulse interval between 100 and 600 ms, and applied to a single presynaptic neuron. (B) Dynamics of the excitatory conductance to a GPe-TI neuron during stimulation for the three synaptic types. (C) Membrane potential dynamics of the GPe-TI neuron during stimulation.

Before implementing the synaptic rules in the full BG networks, the dynamics of each of the three synaptic types were tested on a single GPe-TI neuron receiving DBS pulses from the axon of a single presynaptic STN neuron (Fig 5). For the depression-dominant synapse, the STP variables D (depression) and F (facilitation) reach steady state in a shorter time than for the other two synaptic types (Fig 5A). For both facilitation-dominant and pseudo-linear synapses there was a transient increase in conductance (larger in facilitation-dominant neurons) before reaching a steady state after approximately 300 ms. Depression-dominant synapses were those that exhibit the lowest peak and shortest peak (100 ms) and reached then the lowest steady-state conductance (Fig 5B), thereby limiting the effect of stimulation on postsynaptic neurons (Fig 5C).

### Modeling of dopamine depletion

Dopaminergic degeneration in PD significantly disrupts striatal function by altering the activity of MSNs, which constitute most striatal neurons and are key components of the BG circuitry. Experimental studies have demonstrated that dopamine depletion alters the excitability of projection neurons in a receptor-specific manner, leading to suppressed activity in D1 neurons and enhanced activity in D2 neurons [103,104]. To simulate Parkinsonian condition, we directly implemented the increase in D2 neuron activity by modulating the rate of its external excitatory input. Furthermore, due to the inhibitory nature of projections from D2 to D1 neurons, increased D2 activity resulted in a decreased firing rate of D1 neurons. The intensity of the external input rate towards the D2 population was modeled as:


$$v_{ext}\left(D_{d}\right)=\;v_{ext,\text{0}}(\text{0}.\text{3}D_{d}+\;\text{0}.\text{7}\text{5})$$ (8)

where $v_{ext,\text{0}}$ is the reference value of input rate (see Table 2) and $D_{d}$ is the parameter regulating the severity of the condition of dopamine depletion. The higher is $D_{d}$, the more is dopamine depletion. Specifically, $D_{d}=\text{0}.\text{1}\text{6}\text{6}$ and $D_{d}=\text{0}.\text{5}$ were used for simulating the behavior of the BG in healthy and Parkinsonian condition, respectively.

PD is known to induce synaptic alterations both in the striatal and subthalamo-pallidal loops [69,105,106]. To verify that the results of this manuscript do not depend on the specific modeling of dopamine depletion, we repeated part of the analysis (the ones of Fig 1B and Fig 3D) using an alternative approach to modeling dopamine depletion in the Parkinsonian condition (S6 Fig). Specifically, we incorporated the synaptic alterations described in Lindahl and Kotaleski study [69] (Table 5). Mathematically, in pathological conditions these alterations modify the model parameters *p* by multiplying them by a factor $\text{1}+\beta_{p}\;\phi (\alpha_{d})$ with respect to the healthy case, where $\beta_{p}$ controls the parameter-specific variation and $\phi \left(\alpha_{d}\right)$ regulates the global level of dopamine depletion. In their work, for the Parkinsonian consdition, $\phi (\alpha_{d})$ was set to –0.8.

**Table 5:** Parameters for synaptic alterations with dopamine depletion.

Parameter	Scaling factor in dopamine depletion
$\beta_{g_{inh}}^{FSN-FSN}$	-1.27
$\beta_{g_{inh}}^{GPe\_TA-FSN}$	-0.53
$\beta_{g_{inh}}^{GPe-GPe}$	-0.83
$\beta_{g_{inh}}^{D\text{2}-GPe\_TI}$	-0.83
$\beta_{g_{exc}}^{STN-GPe}$	-0.45
$\beta_{conn.\;prob.}^{FSN-D\text{2}}$	-0.90
$\beta_{g_{inh}}^{D\text{1}/D\text{2}-D\text{1}/D\text{2}}$	0.88
$\beta_{conn.\;prob.}^{D\text{1}/D\text{2}-D\text{1}/D\text{2}}$	0.88
$\beta_{inh}^{GPe\_TA-D\text{1}}$	-1.22
$\beta_{g_{inh}}^{GPe\_TA-D\text{2}}$	-1.15
$\beta_{g_{exc}}^{GPe\_TI-STN}$	-0.24

Because the two modeling approaches differ, a rescaling of $\phi (\alpha_{d})$ was required to obtain the same level of beta synchronization in the absence of DBS. For this reason, we performed a random grid search in which we simulated the network without DBS for 500 different combinations of $\left(D_{d},\phi (\alpha_{d})\right)$. Importantly, we did not alter the relative proportions of the parameter changes (i.e., the βₚ values); instead, we modified only the overall scaling of these changes. Then, for each simulation, we computed the beta-band power. Using this procedure, we identified several combinations that produced levels of beta synchronization comparable to the original model. We then selected an intermediate combination, $D_{d}=\text{0}.\text{3}\;$ and $\phi (\alpha_{d})=-\text{0}.\text{4}$, to perform the DBS analysis on beta power and the PLV between STN and D2 firing rates.

### Modeling of DBS effect

In the present work, we assumed that STN-DBS directly affects only a subset of STN neurons, with the proportion of affected neurons increasing with stimulation intensity. To simulate this effect, the spike trains propagating along the axons of these neurons were replaced with artificial spike trains at the DBS frequency. Let N be the number of STN neurons. For a given stimulation intensity, a fraction (α) of STN neurons, were considered to be stimulated and their axonal outputs were overridden by DBS spike trains $t_{DBS}$, while the remaining axons preserve their natural spike times $t_{STN}$. This modeling approach is motivated by experimental studies investigated the impact of DBS on neuronal behavior. One group of studies explored the target area in which DBS was injected but used different approaches to cope with stimulus artifact; Some of them reported a reduction in neuronal activity [24] and some an increased activity of neurons in the target area [107]. A second group of studies examined downstream structures and found that DBS activates projections from target area [27]. These findings support the hypothesis that DBS exerts both inhibitory and excitatory effect [29]; That is, it could hyperpolarize cell body while still excite an action potential at the axon initial segment. For each fraction of selected neurons, four fixed BG network realizations were used. In each configuration, we gradually increased the fraction of stimulated neurons. For the 2% fraction, STN neurons were selected randomly for stimulation. As the fraction of stimulated neurons increased, the previously selected neurons were retained, and the additional required neurons were chosen randomly from the remaining population. Although this approach does not capture the spatial spread of activation around the electrode, it provides a simplified phenomenological way to mimic the progressive recruitment of neurons with increasing stimulation intensity starting from a seed population proximal to the position of a stimulating contact site in the STN.

In all simulations, DBS was modeled as rectangular monophasic pulses with a total stimulation duration of 6 seconds. The fraction of stimulated neurons (α) was systematically varied to mimic different stimulation intensities. First, results are presented for an inter-pulse interval of $t_{DBS}=\text{7}\;ms$ (corresponding to a stimulation frequency of 143 Hz) to establish baseline effects. Subsequently, additional analyses were performed by systematically varying the DBS frequency to explore its impact on BG network dynamics.

### Spectral and synchronization analysis

The activity of each population was considered as its instantaneous population firing rate, computed as the total number of spikes in population over time bins of one millisecond. Welch method (windows = 2000 ms, overlap = 50%) was used to compute the PSD of the activity.

To quantify the intensity of oscillations in a specific frequency band $\left[f_{\text{1}}\;f_{\text{2}}\right]$, for population i within the BG network, we computed the mean spectral power as:


$$\text{Mean }{PSD}_{i}=\frac{\text{1}}{f_{\text{2}}-f_{\text{1}}}\int_{f_{\text{1}}}^{f_{\text{2}}}P_{i}(f)df$$ (9)

where $P_{i}(f)$ is the PSD of the nucleus activity. Specifically, we used (12–30 Hz) and (30–150 Hz) as the frequency bands for beta and gamma oscillations respectively. This measurement is biased due to the presence of constant activity (no oscillations). To eliminate this bias, we considered the corrected quantity as:


$${PSD}_{i}=\text{ Mean }{PSD}_{i}-Q(N_{i},v_{\text{0},i})$$ (10)

where $Q(N_{i},v_{\text{0},i})$ is the spectral power of an auxiliary population with the same number of neurons and constant mean activity in the nucleus ($i.e,N_{i},v_{\text{0},i}$). The fictitious activity of this auxiliary population has been estimated through the binomial distribution. In other words, the number of spiking neurons in each bin ${\left\{n_{j}^{i}\right\}}_{j}$ for the auxiliary population is given by independent extractions from a binomial distribution:


$$p\left(n_{j}^{i};N_{i},v_{\text{0},i}\right)=B\left(n_{j}^{i};N_{i},v_{\text{0},i}\right)=(\begin{array}{c} N_{i}\\ n_{j}^{i} \end{array}){v_{\text{0},i}}^{n_{j}^{i}}{\left(\text{1}-v_{\text{0},i}\right)}^{N_{i}-n_{j}^{i}}$$ (11)

The synchronization of STN-GPe loop and GPe-striatum loop is responsible for beta exaggeration [23]. We used two approaches to measure the synchronization between the two loops in different conditions of BG. The first one is comparing the mean frequency of beta oscillations in D2 and STN as representatives of the two loops:


$$\text{Mean }f_{i}=\frac{\text{1}}{\int_{\text{1}\text{2}}^{\text{3}\text{0}}P_{i}(f)df}\int_{\text{1}\text{2}}^{\text{3}\text{0}}{fP}_{i}(f)df$$ (12)

The second approach is to use PLV between the activities of STN and D2 as a measurement of the intensity of synchronization. PLV is defined as:


$$\text{PLV}=\left|\frac{\text{1}}{N}\sum {\mathrm{\backslash nolimits}}_{n=\text{1}}^{N}e^{i(\varphi_{STN}(n)-\varphi_{D_{\text{2}}}(n))}\right|$$ (13)

where $\varphi_{STN}(.)$ and $\varphi_{D_{\text{2}}}(.)$ represent the phase time series of the beta range activities of STN and D2, respectively. The phase time series are computed as:


$$\varphi_{x}=\text{mod}(\text{angle}\left(\text{Hilbert}(x)),\text{2}\pi \right))$$ (14)

where x∈{STN, D2} represents the beta range activities of STN and D2 and is computed by bandpass filtering of STN and D2 activities with cut off frequencies of 11 and 31 Hz.

### Numerical methods

The code for simulating the BG network has been developed in Python using ANNarchy [108]. Post analysis of the simulation results has been implemented in Python as well. The fourth-order Runge-Kutta approach with a fixed time step h = 0.04 ms was used to numerically integrate the equations that describe the development of each neuron in the simulated network. The duration of each simulation was fixed to 6000 ms and the first 2000 ms was eliminated as of the transition state. For each case, 4 simulations were performed to estimate the standard error of computed quantities.

## Supporting information

S1 Fig Effect of Stimulation duration on STN beta power. (PDF)

S2 Fig Robustness of DBS efficacy to changes in synaptic plasticity time scales. (PDF)

S3 Fig Contribution of individual STP synapse types to DBS efficacy. (PDF)

S4 Fig Effect of DBS intensity on STN activity in BG network model without plasticity. (PDF)

S5 Fig Effects of STP at STN-to-GPe projections on DBS. (PDF)

S6 Fig DBS effects on BG network model with dopamine-depletion-induced synaptic alterations. (PDF)

S7 Fig Temporal modulation of BG network activity under STN-DBS. (PDF)
