# Supplementary material for: In silico model of basal ganglia deep brain stimulation in Parkinson’s disease captures range of effective parameters for pathological beta power suppression
Source: PLoS Comput Biol. 2026 Feb 11;22(2):e1013280. doi: 10.1371/journal.pcbi.1013280 (PMC12916059; doi:10.1371/journal.pcbi.1013280)
Supplement: S1 Fig — (PDF) [file pcbi.1013280.s001.pdf]

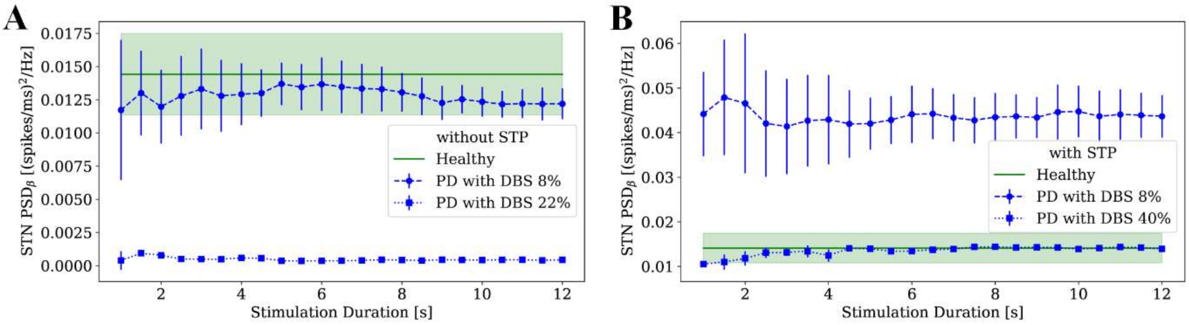

S1 Fig. Effect of Stimulation duration on STN beta power. Variation of STN beta power in relation to stimulation duration is shown for BG network model without plasticity (A, DBS intensities: 8%, 22%) and with plasticity (B, DBS intensities: 8%, 40%). DBS was delivered with 7 ms inter-pulse interval. STN beta power in healthy (green) conditions are also presented. For each condition, four BG network realizations were simulated, and the mean STN beta power is plotted. For the healthy conditions (green), the shaded areas around the means represent the standard error across the four simulations. For the DBS conditions, standard error across the four simulations is shown using error bars.
