# Supplementary material for: In silico model of basal ganglia deep brain stimulation in Parkinson’s disease captures range of effective parameters for pathological beta power suppression
Source: PLoS Comput Biol. 2026 Feb 11;22(2):e1013280. doi: 10.1371/journal.pcbi.1013280 (PMC12916059; doi:10.1371/journal.pcbi.1013280)
Supplement: S2 Fig — (PDF) [file pcbi.1013280.s002.pdf]

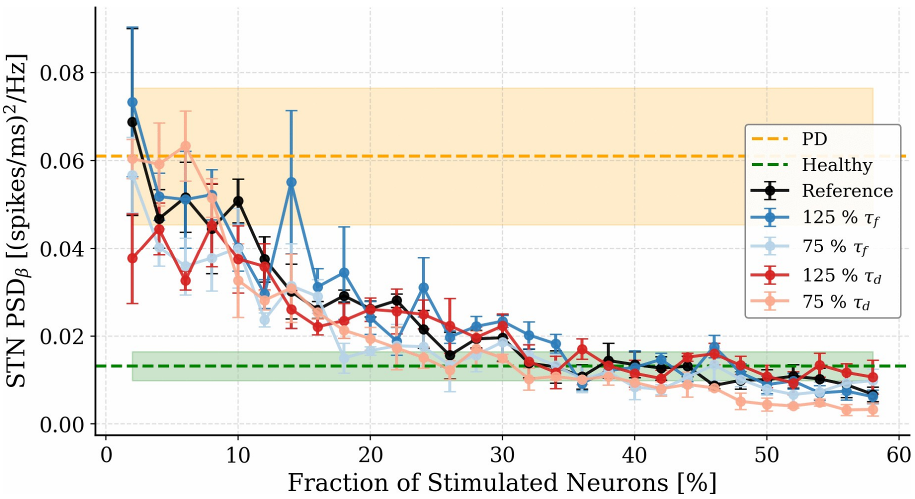

S2 Fig. Robustness of DBS efficacy to changes in synaptic plasticity time scales. Variation of STN beta power in relation to fraction of stimulated neurons is shown for BG network model with different values of synaptic plasticity time scales. DBS was delivered with 7 ms inter-pulse interval. In blue and light blue, the effects of increasing and decreasing the facilitation time scale of all three synapse types by 25%, respectively. In red and orange, the effects of increasing and decreasing the depression time scale of all three synapse types by 25%, respectively. "Reference" (black) refers to the model used in Fig 1B with reference values for synaptic timescales. Healthy and Parkinsonian beta power are shown in green and orange, respectively. For each condition, four BG network realizations were simulated, and the mean STN beta power was plotted. The shaded area around the mean represents the standard error across the four simulations. For the DBS conditions, standard error across the four simulations is shown using error bars.
