# Supplementary material for: In silico model of basal ganglia deep brain stimulation in Parkinson’s disease captures range of effective parameters for pathological beta power suppression
Source: PLoS Comput Biol. 2026 Feb 11;22(2):e1013280. doi: 10.1371/journal.pcbi.1013280 (PMC12916059; doi:10.1371/journal.pcbi.1013280)
Supplement: S3 Fig — (PDF) [file pcbi.1013280.s003.pdf]

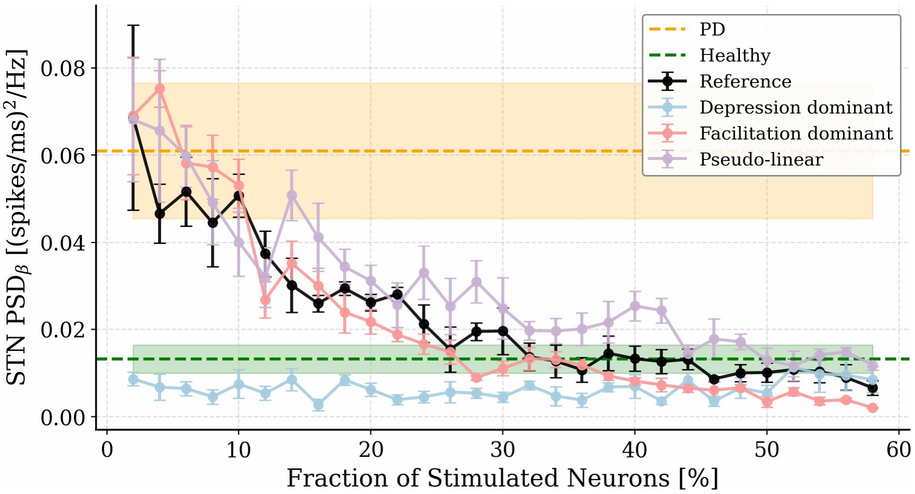

S3 Fig. Contribution of individual STP synapse types to DBS efficacy. Effect of the fraction of stimulated neurons on the effectiveness of DBS with 7 ms inter-pulse interval in suppressing beta oscillations, considering only one synaptic type at a time. Pink: depression-dominant synapses; purple: facilitation-dominant synapses; brown: pseudo-linear synapses. “Reference” (black) refers to the model used throughout the manuscript, which includes all three synaptic types. Healthy and Parkinsonian beta power are shown in green and orange, respectively. For each condition, four BG network realizations were simulated, and the mean STN beta power was plotted. The shaded area around the mean represents the standard error across the four simulations. For the DBS conditions, standard error across the four simulations is shown using error bars.
