# Supplementary material for: In silico model of basal ganglia deep brain stimulation in Parkinson’s disease captures range of effective parameters for pathological beta power suppression
Source: PLoS Comput Biol. 2026 Feb 11;22(2):e1013280. doi: 10.1371/journal.pcbi.1013280 (PMC12916059; doi:10.1371/journal.pcbi.1013280)
Supplement: S4 Fig — (PDF) [file pcbi.1013280.s004.pdf]

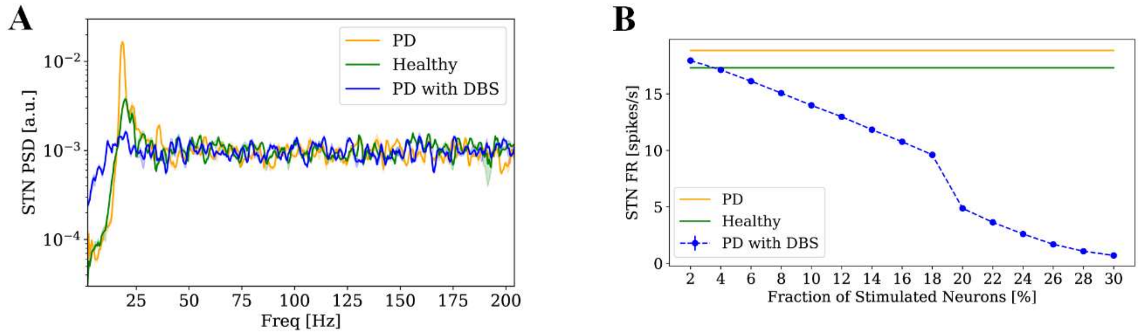

S4 Fig. Effect of DBS intensity on STN activity in BG network model without plasticity. DBS was delivered with 7 ms inter-pulse interval. (A) Normalized PSD (PSD divided by total power) of STN in healthy (green), Parkinsonian (orange) and Parkinsonian with STN-DBS at 22% intensity (blue) conditions in BG network model without plasticity. PSDs are reported as mean and standard error across four simulations. (B) Variation of STN firing rate in relation to fraction of stimulated neurons in BG network model without plasticity. STN firing rate in healthy (green) and Parkinsonian conditions (orange) are also presented. For each condition, four BG network realizations were simulated, and results are presented as mean and standard error across four simulations.
