# Supplementary material for: In silico model of basal ganglia deep brain stimulation in Parkinson’s disease captures range of effective parameters for pathological beta power suppression
Source: PLoS Comput Biol. 2026 Feb 11;22(2):e1013280. doi: 10.1371/journal.pcbi.1013280 (PMC12916059; doi:10.1371/journal.pcbi.1013280)
Supplement: S5 Fig — (PDF) [file pcbi.1013280.s005.pdf]

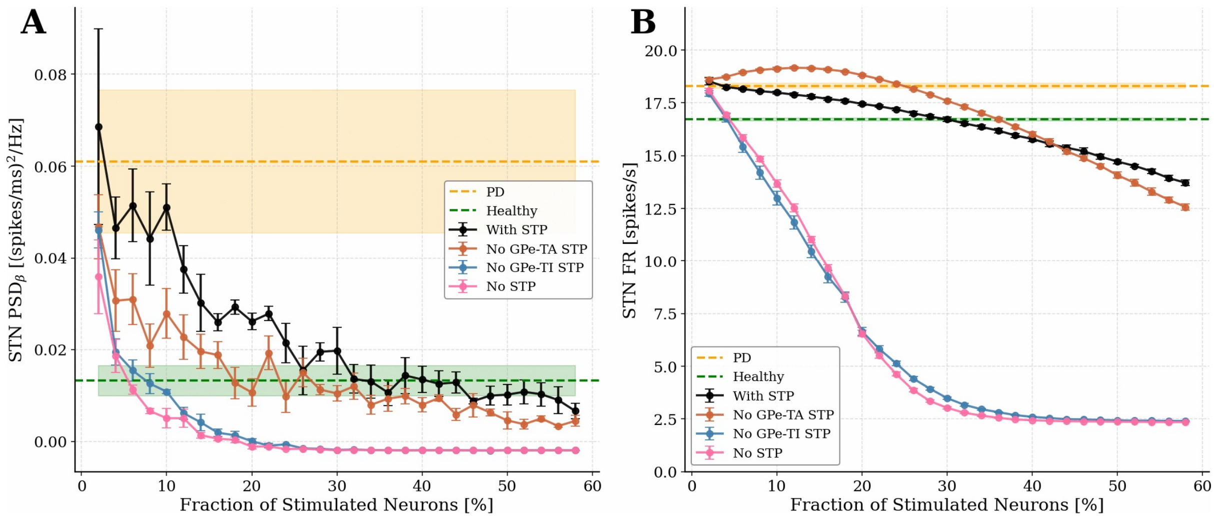

S5 Fig. Effects of STP at STN-to-GPe projections on DBS. DBS was delivered with 7 ms inter-pulse interval. (A) Effect of the fraction of stimulated neurons on the effectiveness of DBS in suppressing beta oscillations when STP is removed from specific projections. Black: STP present in all STN-to-GPe synapses; brown: STP present only in the GPe-TI projection; blue: STP present only in the GPe-TA projection; pink: STP removed entirely from the model. Healthy and Parkinsonian beta power are shown in green and orange, respectively. For each condition, four BG network realizations were simulated, and the mean STN beta power was plotted. The shaded area around the mean represents the standard error across the four simulations. For the DBS conditions, standard error across the four simulations is shown using error bars. (B) Effect of the fraction of stimulated neurons on STN firing rate when STP is removed from specific projections. For each condition, four BG network realizations were simulated, and results are presented as mean and standard error across four simulations.
