# Supplementary material for: In silico model of basal ganglia deep brain stimulation in Parkinson’s disease captures range of effective parameters for pathological beta power suppression
Source: PLoS Comput Biol. 2026 Feb 11;22(2):e1013280. doi: 10.1371/journal.pcbi.1013280 (PMC12916059; doi:10.1371/journal.pcbi.1013280)
Supplement: S6 Fig — (PDF) [file pcbi.1013280.s006.pdf]

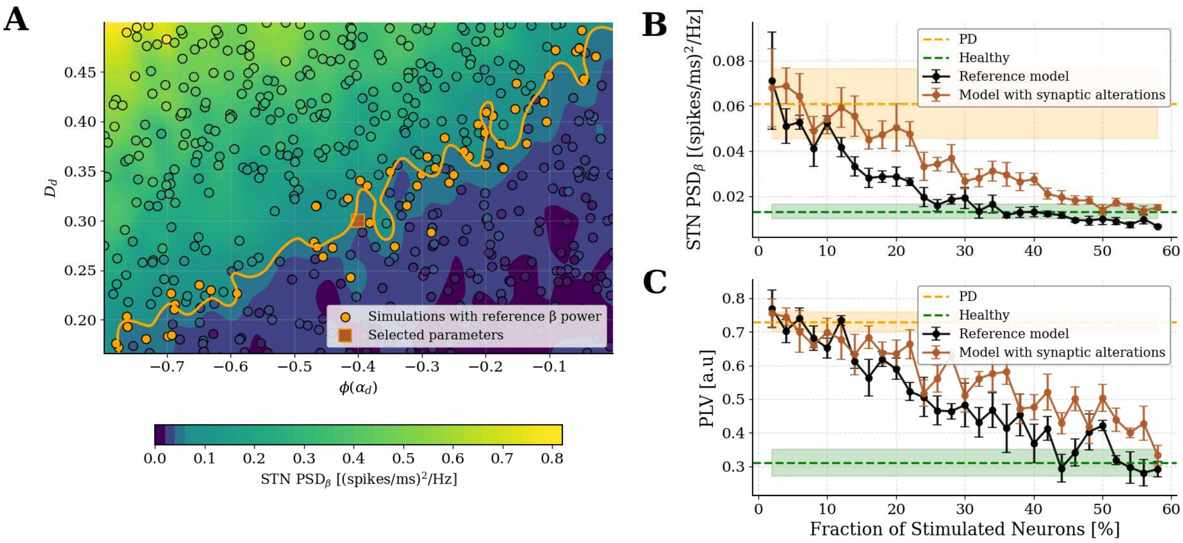

S6 Fig. DBS effects on BG network model with dopamine-depletion-induced synaptic alterations. (A) STN beta power for 500 different combinations of the parameters D_d (modeling the increased input to the D2 population under dopamine-depleted conditions) and $\phi(\alpha_d)$ (modeling the synaptic alterations following dopamine depletion). Combinations whose beta power falls within one standard error of the reference value, calculated across four simulations of the reference model with $D_d = 0.5$ and $\phi(\alpha_d) = 0$ (i.e., no synaptic alterations), are highlighted in orange. The parameters selected for the model with synaptic alterations ($D_d = 0.3$ and $\phi(\alpha_d) = -0.4$) are highlighted by a brown square. The effects of DBS on the reference model (black, $D_d = 0.5$, $\phi(\alpha_d) = 0$) and the model with synaptic alteration (brown, $D_d = 0.3$, $\phi(\alpha_d) = -0.4$) are compared for STN beta power (B) and PLV between D2 and STN (C) as a function of the fraction of stimulated neurons. Healthy and Parkinsonian conditions for the reference model are shown in green and orange, respectively. For each condition, four BG network realizations were simulated, and the mean value is reported. The shaded area around the mean represents the standard error across the four simulations. For the DBS conditions, standard error across the four simulations is shown using error bars.
