# Supplementary material for: In silico model of basal ganglia deep brain stimulation in Parkinson’s disease captures range of effective parameters for pathological beta power suppression
Source: PLoS Comput Biol. 2026 Feb 11;22(2):e1013280. doi: 10.1371/journal.pcbi.1013280 (PMC12916059; doi:10.1371/journal.pcbi.1013280)
Supplement: S7 Fig — (PDF) [file pcbi.1013280.s007.pdf]

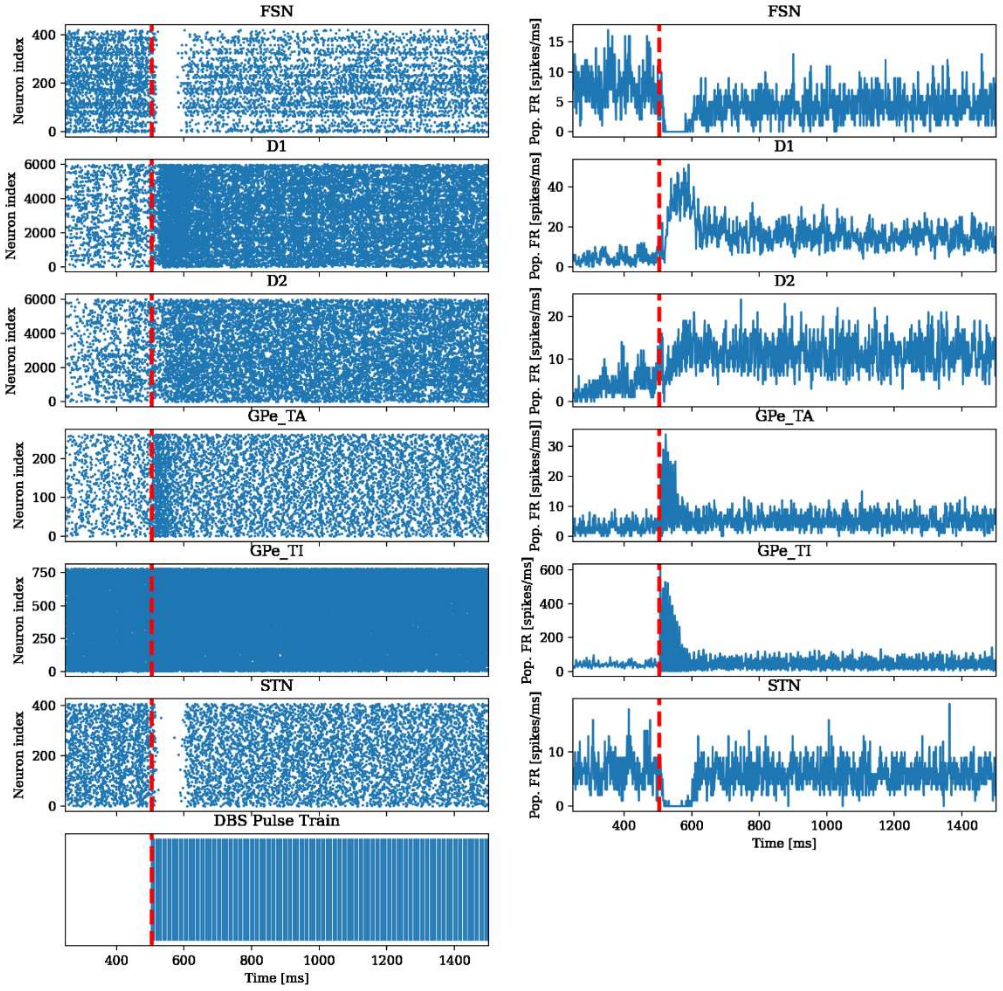

S7 Fig. Temporal modulation of BG network activity under STN-DBS. Raster plots and instantaneous population firing rates of BG network structures in Parkinsonian condition with STN-DBS at 40% intensity, shown alongside the DBS pulse train. DBS onset occurred at 500 ms (indicated with red dashed line), following a Parkinsonian baseline period. DBS was delivered with 7 ms inter-pulse interval.
